# Supplementary material for: Control of seed dormancy and germination by DOG1-AHG1 PP2C phosphatase complex via binding to heme
Source: Nat Commun. 2018 Jun 6;9:2132. doi: 10.1038/s41467-018-04437-9 (PMC5989226; doi:10.1038/s41467-018-04437-9)
Supplement: Supplementary file 1 — Supplementary Information [file 41467_2018_4437_MOESM1_ESM.pdf]

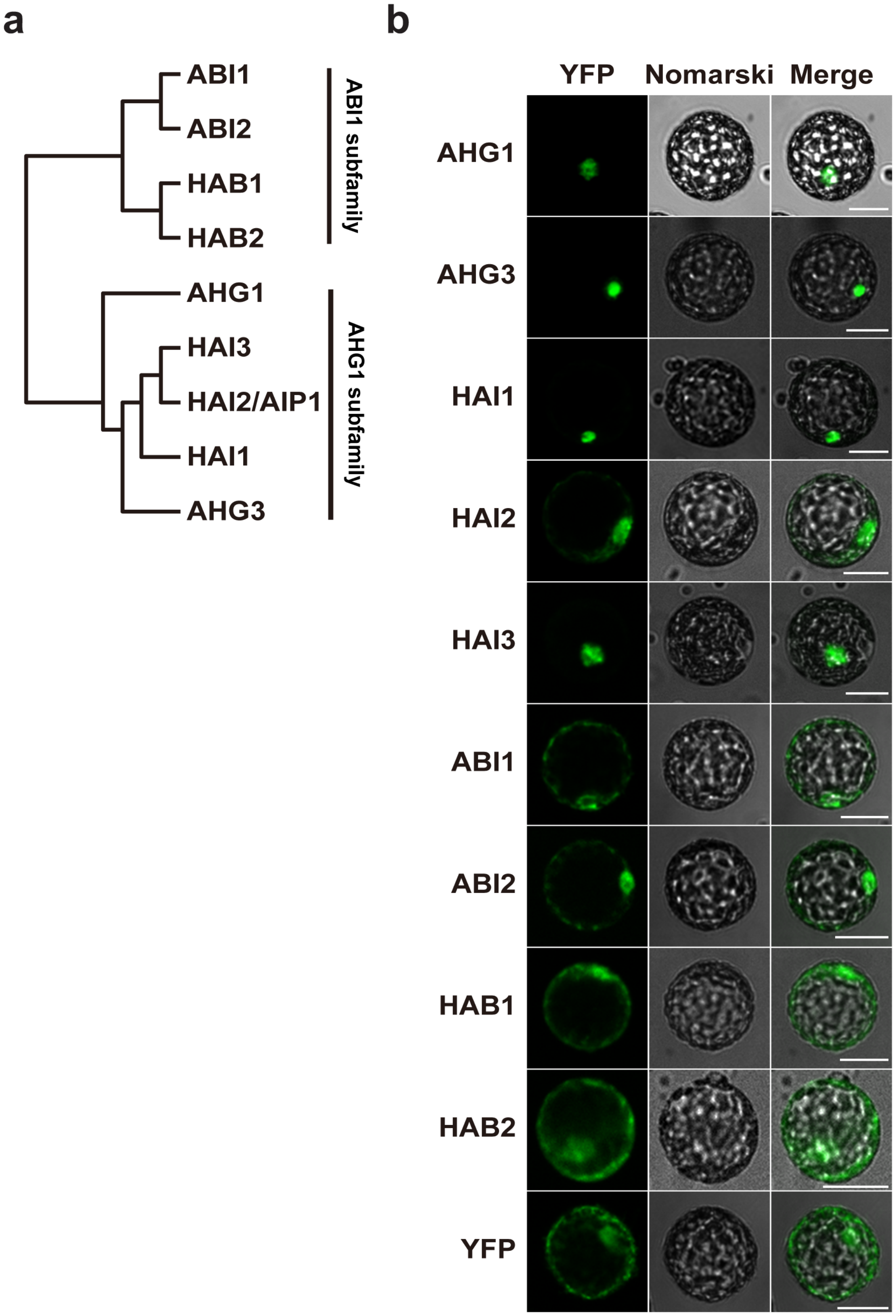

Supplementary Figure 1. The subcellular localization of group A PP2Cs. (**a**) Phylogenetic tree of group A PP2Cs. The phylogenetic analysis was performed by CLUSTAL W. (**b**) Subcellular localization of YFP-PP2Cs in *N. benthamiana* protoplasts. Image columns depict YFP fluorescence of *N. benthamiana* protoplasts expressing YFP-PP2Cs and YFP control. The photos were YFP, Nomarski and merged images. Scale bars, 20μm.

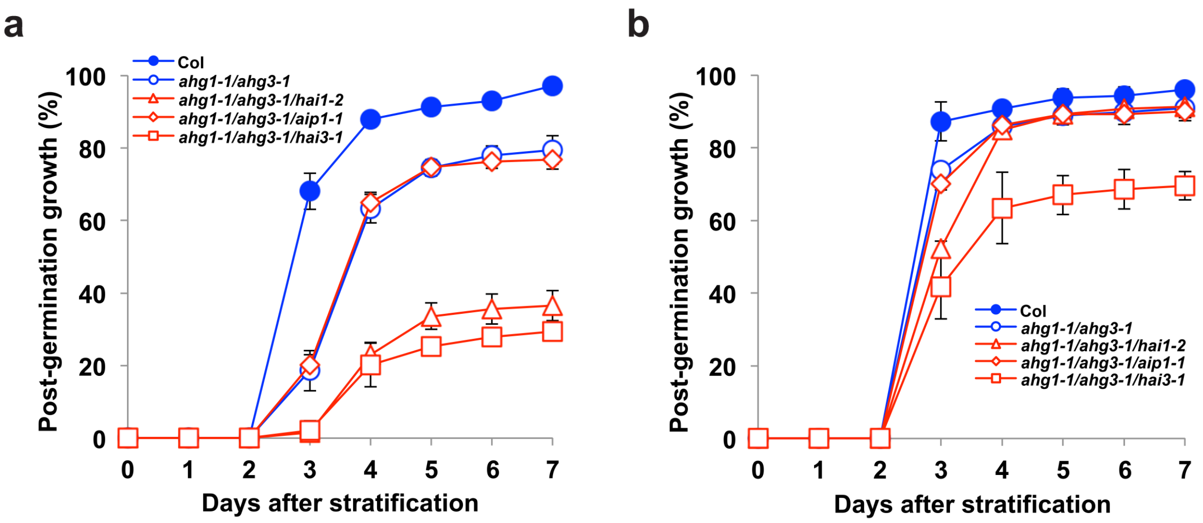

Supplementary Figure 2. The triple mutants of *PP2C* show an increased seed dormancy. (**a**,**b**) Post-germination growth efficiencies of *pp2c* double and triple mutant lines (*ahg1-1ahg3-1*, *ahg1-1ahg3-1hai1-2*, *ahg1-1ahg3-1aip1-1*, and *ahg1-1ahg3-1hai3-1*) and wild type were examined with stratification for 0 days (a) or 4 days (b). Error bars show s.d. of three independent experiments using the same seed batch (a,b).

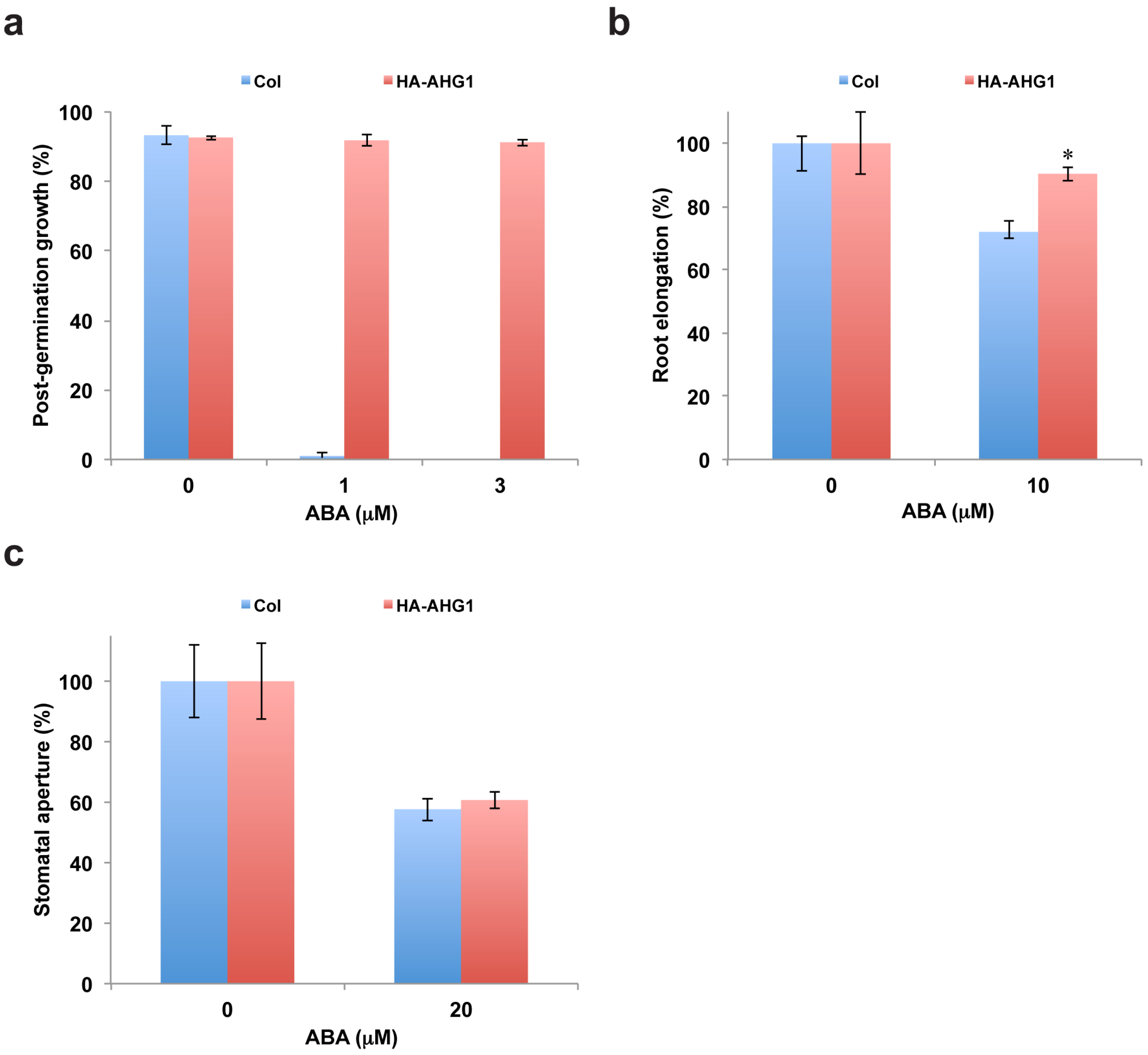

Supplementary Figure 3. Overexpression of HA-AHG1 causes ABA insensitivity in seed germination and root growth response. (**a**) Post-germination growth efficiencies of overexpressing HA-AHG1 line and wild type Col in the presence of various concentrations of ABA at 7 days after stratification. Error bars show s.d. of three independent experiments using the same seed batch. (**b**) ABA dependent root growth responses of overexpressing HA-AHG1 line and wild type Col. Seedlings were germinated and grown on hormone-free MS plates for 5 days and then transferred to MS plates with or without 10 μM ABA. Root length was measured 4 days after transfer. Error bars show s.e.m. of three independent experiments. An asterisk indicates significant difference between the corresponding values (*$P<0.05$; Tukey-Kramer test). (**c**) ABA-induced stomatal closure in overexpressing HA-AHG1 line and wild type Col. The epidermal tissues isolated from dark-adapted 4- to 6-week-old plants were incubated in basal buffer (5 mM MES-BTP pH 6.5, 50 mM KCl, and 0.1 mM $CaCl_2$). Pre-illuminated epidermal tissues were incubated under light (red light at 50 μmol m^{-2} s^{-1} and blue light at 10 μmol m^{-2} s^{-1}) for 2.5 h with or without 20 μM ABA. Error bars show s.e.m. of three independent experiments (35 stomata per experiment and condition). No significant difference between the corresponding values ($P>0.05$; Tukey-Kramer test).

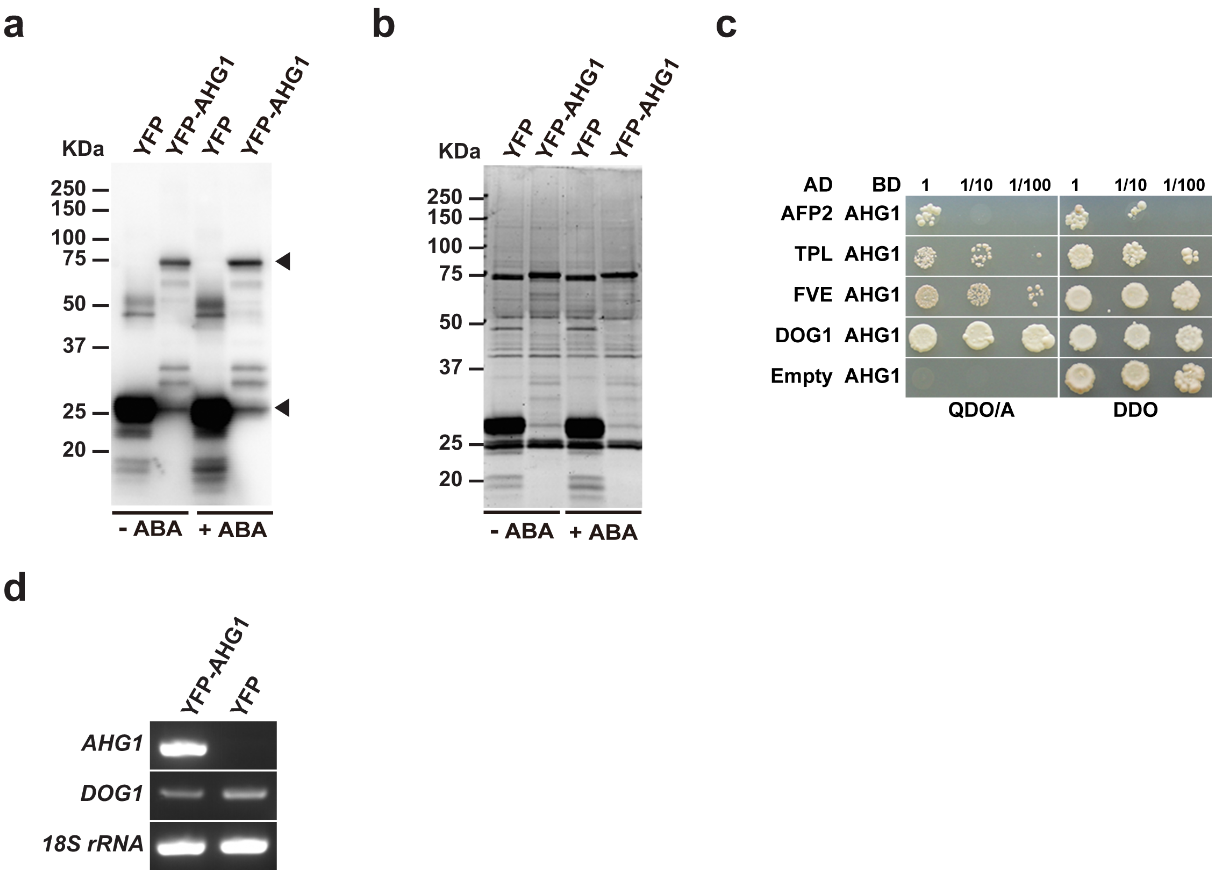

Supplementary Figure 4. Identification of AHG1-interacting proteins in *Arabidopsis*. (**a**) Western blot analysis of YFP and YFP-AHG1 proteins in *Arabidopsis* plants overexpressing YFP or YFP-AHG1 treated with or without ABA after GFP affinity column purification with anti-GFP antibody. Arrow heads predicted YFP and YFP-AHG1 bands. (**b**) YFP-AHG1 and associated proteins purified form *Arabidopsis* plants overexpressing YFP or YFP-AHG1 treated with or without ABA. Eluates from YFP and YFP-AHG1 purifications were analyzed and visualized by SDS-PAGE gel staining with Oriole. (**c**) Validation of the physical interaction between AHG1 and AHG1-interacting proteins by the yeast two-hybrid assay. Y2H Gold cells transformed with GAL4BD-AHG1 and GAL4AD-AHG1-interacting proteins as indicated and were spotted onto DDO (Double dropout medium: SD/-Leu/-Trp) and QDO/A (Quadruple dropout medium: SD/-Ade/-His/-Leu/-Trp supplemented with Aureobasidin A) medium agar plates for 7 days after inoculation. (**d**) RT-PCR analysis of *AHG1* and *DOG1* expression in overexpressing YFP-AHG1 and control YFP lines. Total RNA was isolated from three-week-old seedlings grown on MS plate were incubated for 2h in water. DNA fragments for *AHG1* and *DOG1* were amplified for 30 PCR cycles by use of gene specific primers. *18s rRNA* was amplified for 20 PCR cycles and used as an internal control.

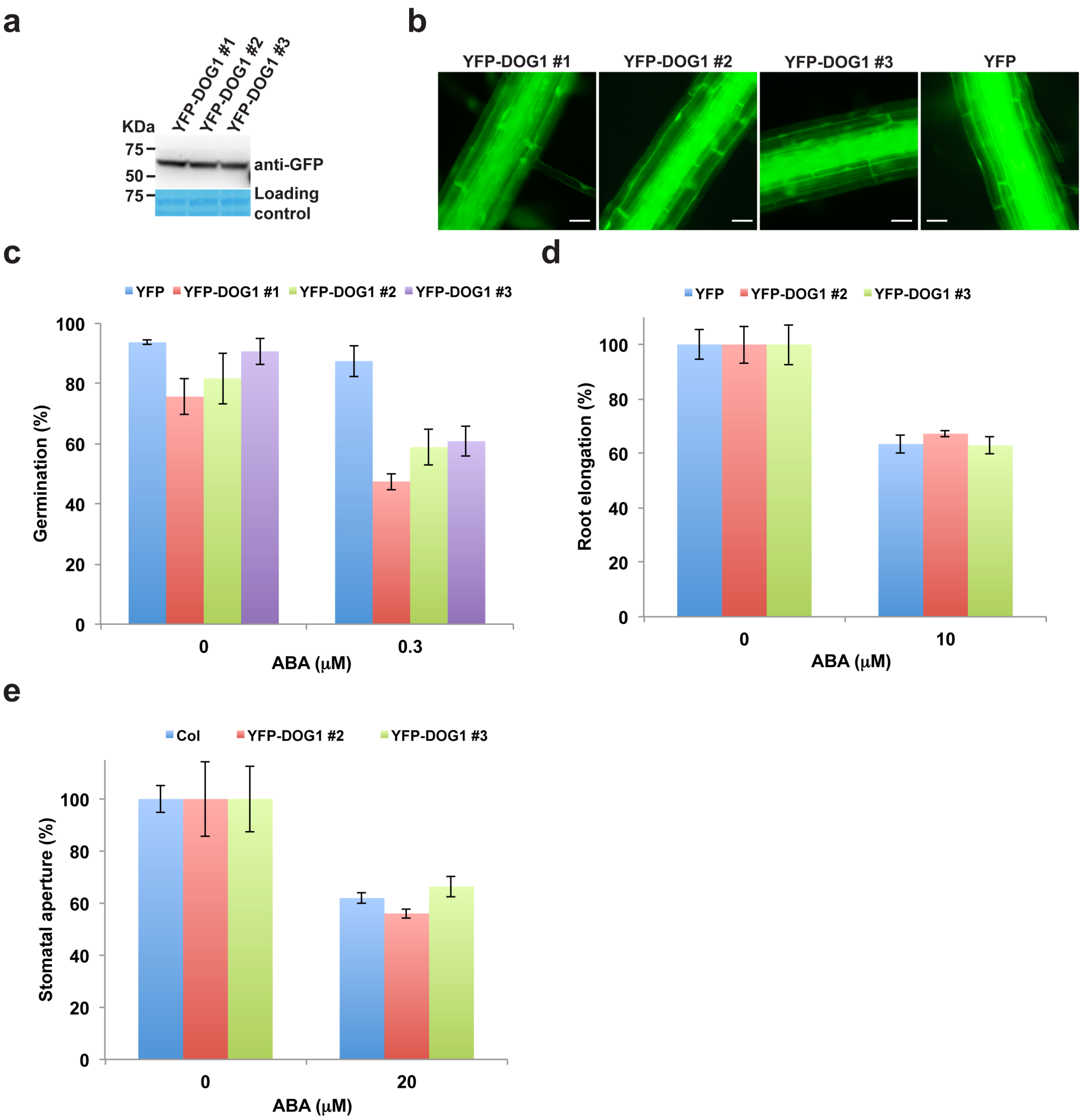

Supplementary Figure 5. Overexpression of YFP-DOG1 shows ABA hypersensitivity in seed germination. (**a**) Western blot analysis of YFP-DOG1 protein levels in *Arabidopsis* plants overexpressing YFP-DOG1. Total protein was isolated from seedlings grown on MS plate for 7 days. A nonspecific band stained at approx. 70 kDa is used as a loading control. (**b**) The subcellular localization of *Arabidopsis* plants overexpressing YFP-DOG1 and YFP. Scale bars, 20µm. (**c**) Germination efficiencies of overexpressing YFP-DOG1 and control YFP lines were treated with or without 0.3 µM ABA at 3 days after stratification. Error bars show s.d. of three independent experiments using the same seed batch. (**d**) ABA dependent root growth responses of overexpressing YFP-DOG1 and control YFP lines. Seedlings were germinated and grown on hormone-free MS plates for 5 days and then transferred to MS plates with or without 10 µM ABA. Root length was measured 4 days after transfer. Error bars show s.e.m. of three independent experiments. No significant difference between the corresponding values (P>0.05; Tukey-Kramer test). (**e**) ABA-induced stomatal closure in

overexpressing YFP-DOG1 lines and wild type Col. The epidermal tissues isolated from dark-adapted 4- to 6-week-old plants were incubated in basal buffer (5 mM MES-BTP pH 6.5, 50 mM KCl, and 0.1 mM $CaCl_2$). Pre-illuminated epidermal tissues were incubated under light (red light at 50 μmol m^{-2} s^{-1} and blue light at 10 μmol m^{-2} s^{-1}) for 2.5 h with or without 20 μM ABA. Error bars show s.e.m. of three biological replicates (35 stomata per experiment and condition). No significant difference between the corresponding values ($P>0.05$; Tukey-Kramer test).

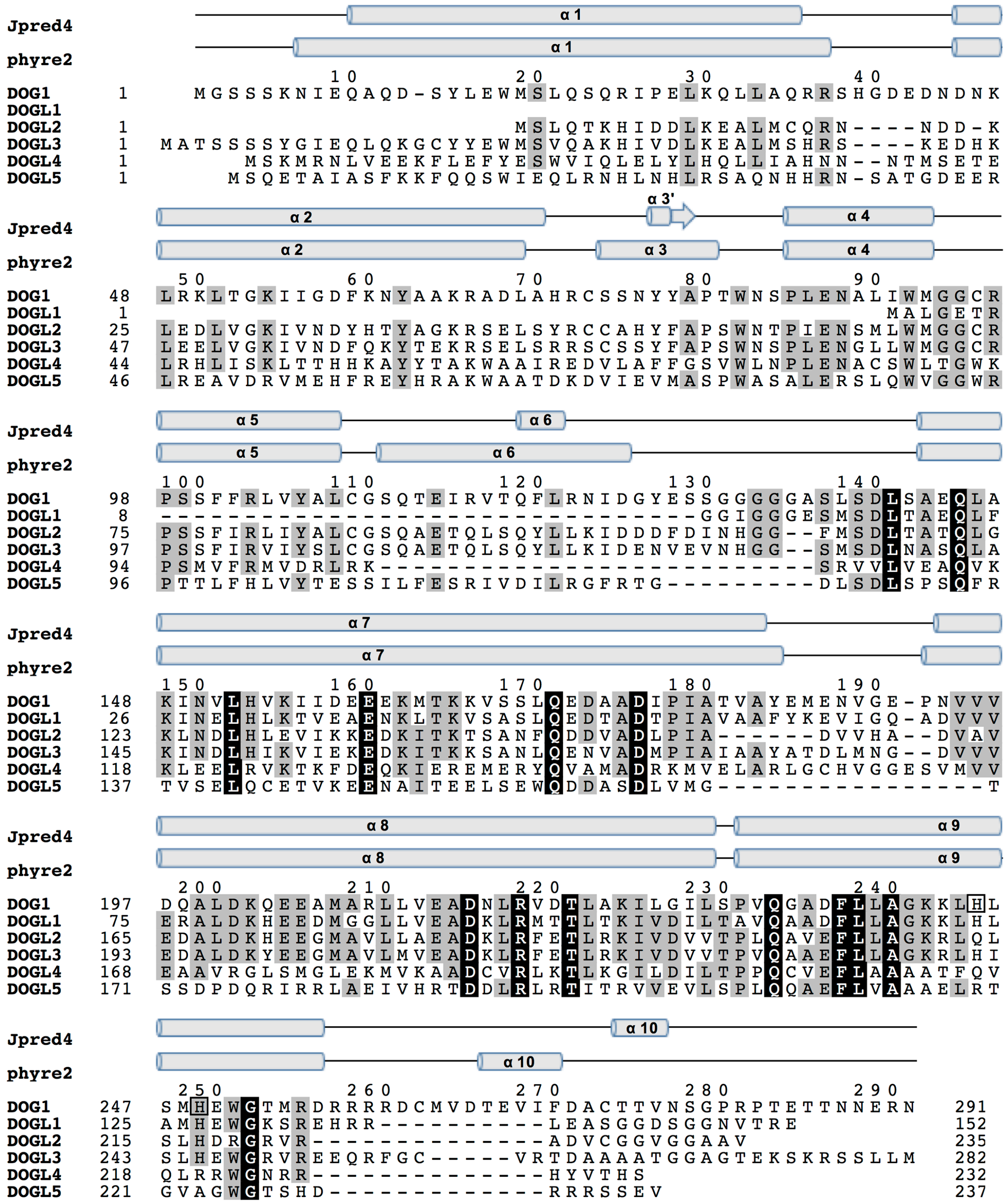

Supplementary Figure 6. Sequence alignment of DOG1/DOGL proteins in *Arabidopsis*. Sequences were aligned using the Clustal W2[1]. The secondary structures of DOG1 predicted by Jpred4 and Phyre2 are shown above the sequence[2, 3]. His245 and His249 residues of DOG1 are boxed.

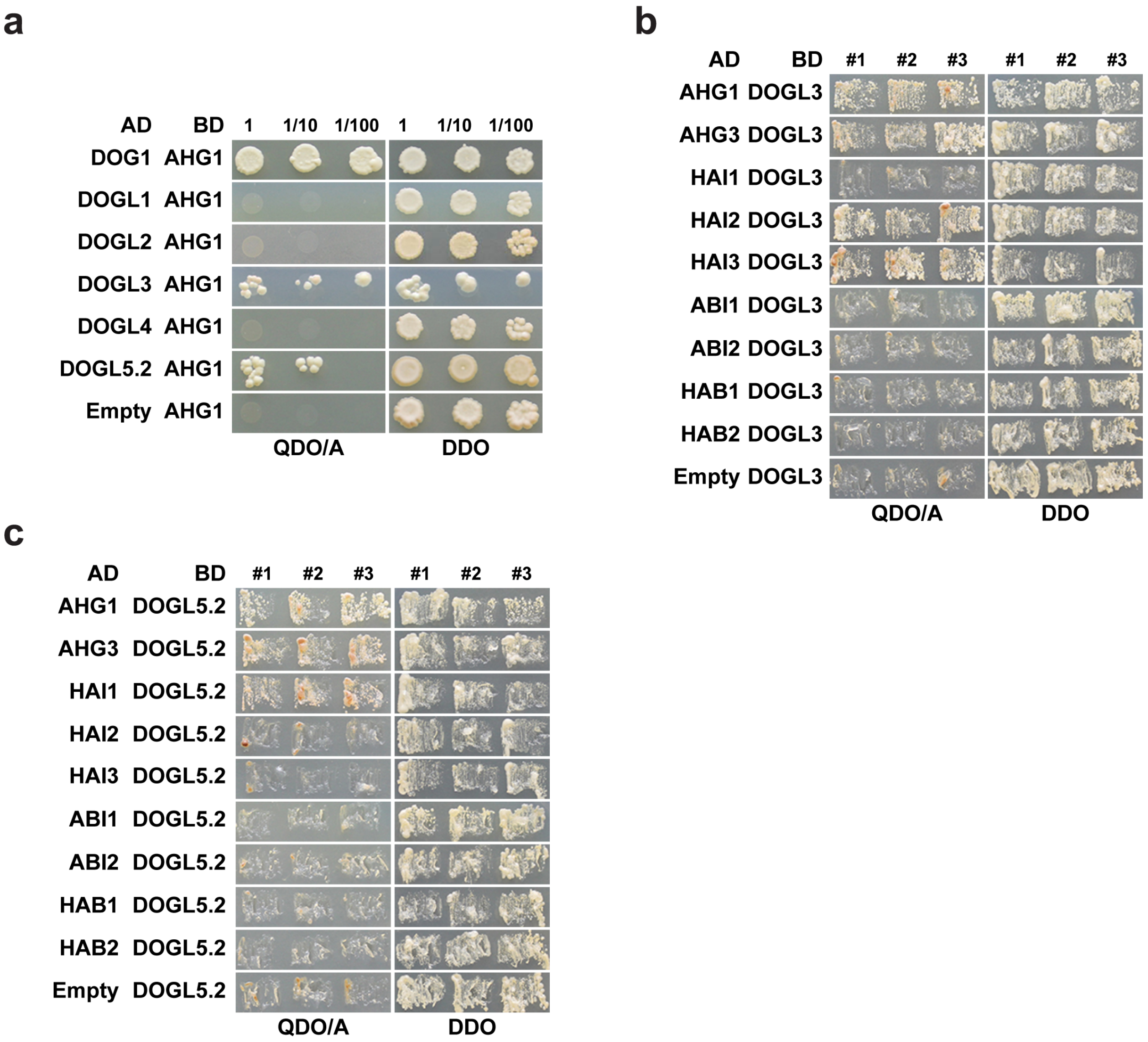

Supplementary Figure 7. Physical interaction of AHG1 with DOG1/DOGLs. (**a**) Yeast-two-hybrid analysis of DOG1/DOGLs with AHG1. Y2H Gold cells transformed with GAL4BD-AHG1 and GAL4AD-DOG1/DOGLs as indicated and were spotted onto DDO (Double dropout medium: SD/-Leu/-Trp) and QDO/A (Quadruple dropout medium: SD/-Ade/-His/-Leu/-Trp supplemented with Aureobasidin A) medium agar plates for 7 days after inoculation. (**b**, **c**) Yeast-two-hybrid analysis of group A PP2Cs with DOGL3 and DOGL5.2. Y2H Gold cells transformed with GAL4BD-DOGL3 (b) or GAL4BD-DOGL5.2 (c) and GAL4AD-PP2Cs as indicated. Three independent transformants were grown onto DDO (Double dropout medium: SD/-Leu/-Trp) and QDO/A (Quadruple dropout medium: SD/-Ade/-His/-Leu/-Trp supplemented with Aureobasidin A) medium agar plates for 7 days.

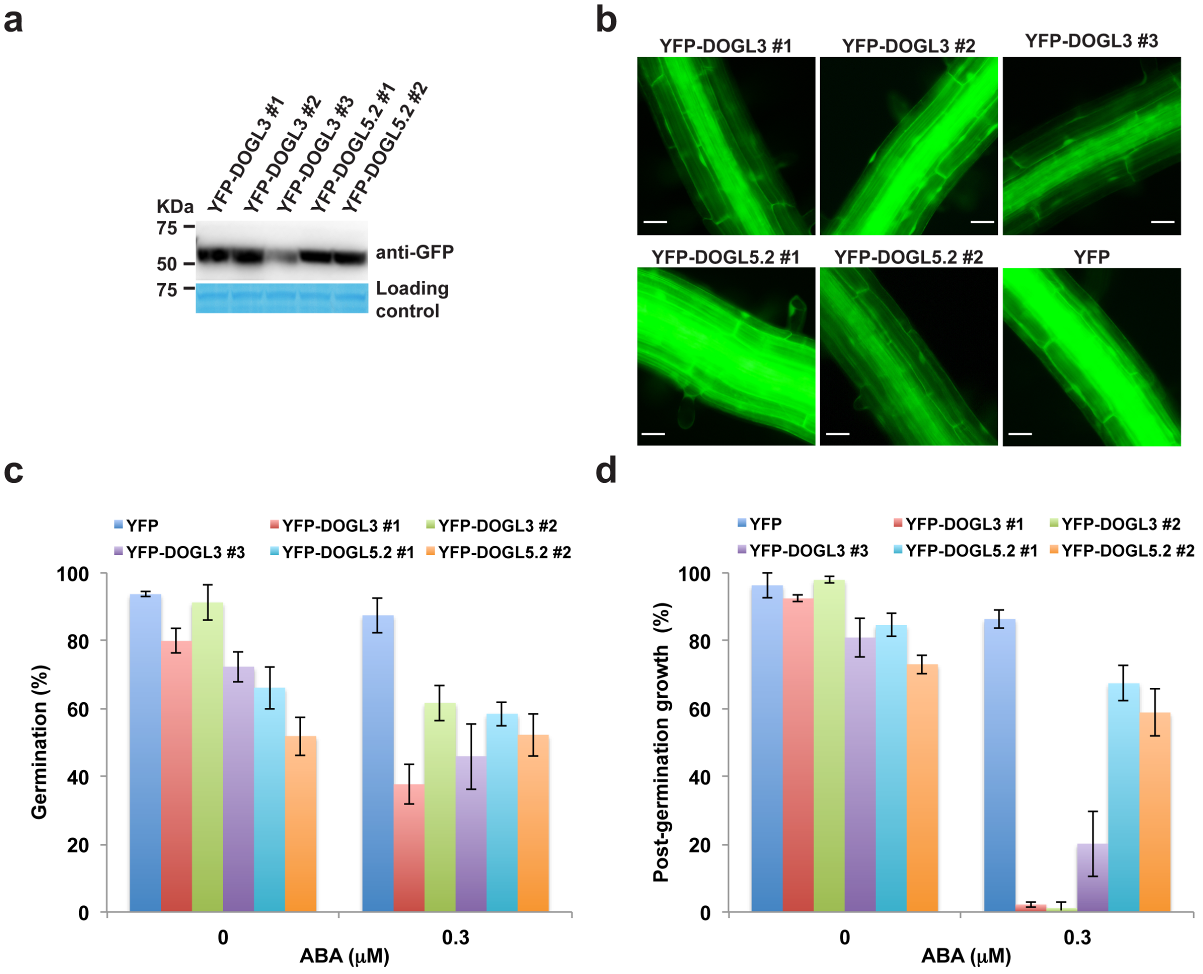

Supplementary Figure 8. Overexpression of YFP-DOGL3 shows ABA hypersensitivity in seed germination. (**a**) Western blot analysis of YFP-DOGL3 and YFP-DOGL5.2 protein levels in *Arabidopsis* plants overexpressing YFP-DOGL3 and YFP-DOGL5.2 lines. Total protein was isolated from seedlings grown on MS plate for 7 days. A nonspecific band stained around 70 kDa is used as a loading control. (**b**) The subcellular localization of *Arabidopsis* plants overexpressing YFP-DOGL3, YFP-DOGL5.2 and YFP. Scale bars, 20μm. (**c**,**d**) Germination efficiencies (c) and post-germination growth efficiencies (d) of overexpressing YFP-DOGL3, YFP-DOGL5.2 and control YFP lines were treated with or without 0.3 μM ABA at 3 days (c) and 7 days (d) after stratification. Error bars show s.d. of three independent experiments using the same seed batch (c,d).

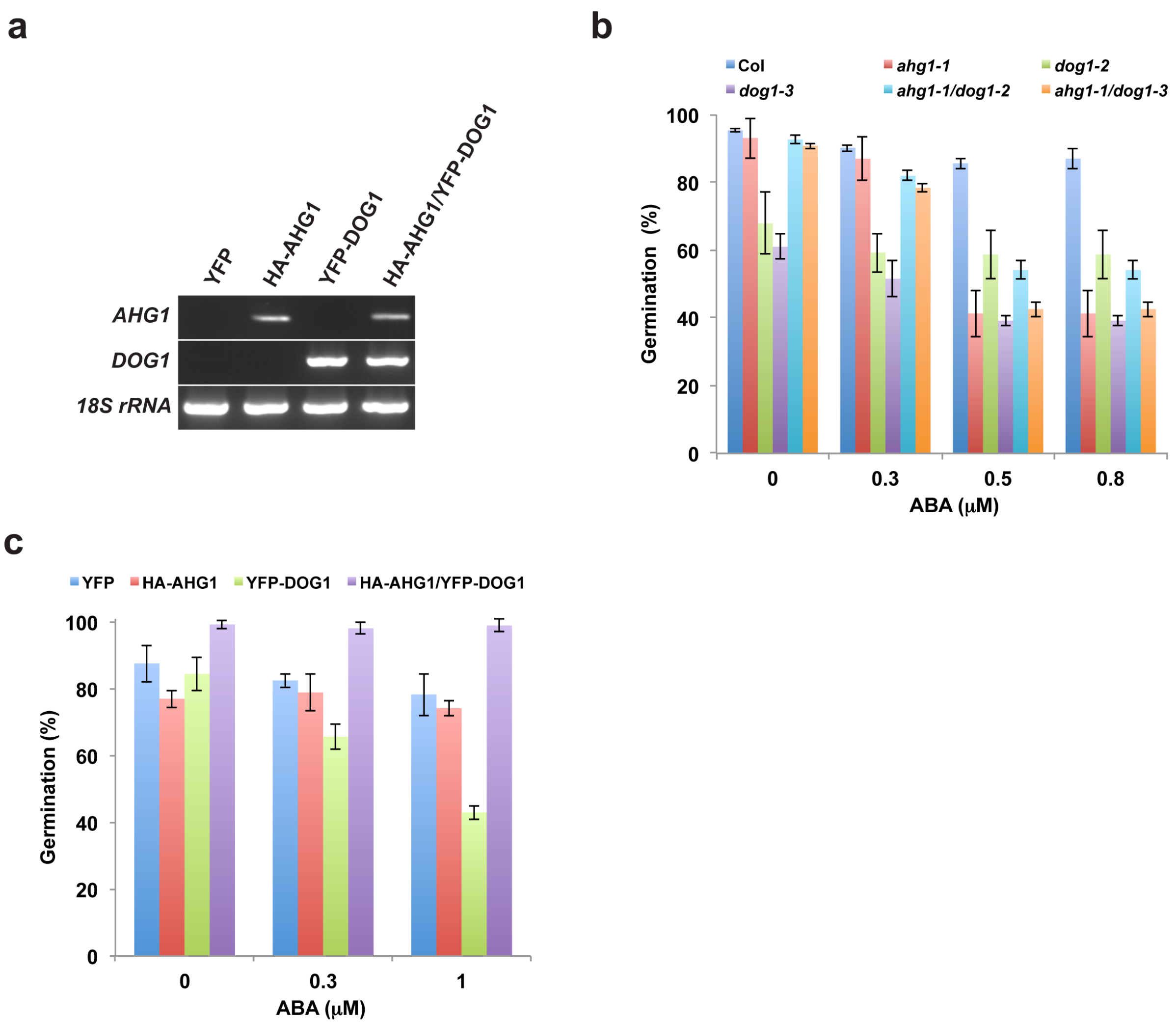

Supplementary Figure 9. Genetic interaction of *AHG1* and *DOG1*. (**a**) RT-PCR analysis of *AHG1* and *DOG1* expression in the double overexpressing (HA-AHG1ox/YFP-DOG1ox), parental (HA-AHG1ox and YFP-DOG1ox) and control YFP lines. Total RNA was isolated from seedlings grown on MS plate for 7 days. The transcripts of the *18s rRNA* gene were used as an internal control. DNA fragments for *AHG1*, *DOG1* and *18s rRNA* were amplified for 20 PCR cycles by use of gene specific primers. (**b**) Germination efficiencies of the single and double mutant lines (*ahg1-1*, *dog1-2*, *dog1-3*, *ahg1-1dog1-2*, and *ahg1-1dog1-3*) and wild type were examined in the presence of various concentrations of ABA at 3 days after stratification. (**c**) Germination efficiencies of the double overexpressing, parental and control YFP lines were examined in the presence of various concentrations of ABA at 3 days after stratification. Error bars show s.d. of three independent experiments using the same seed batch (b,c).

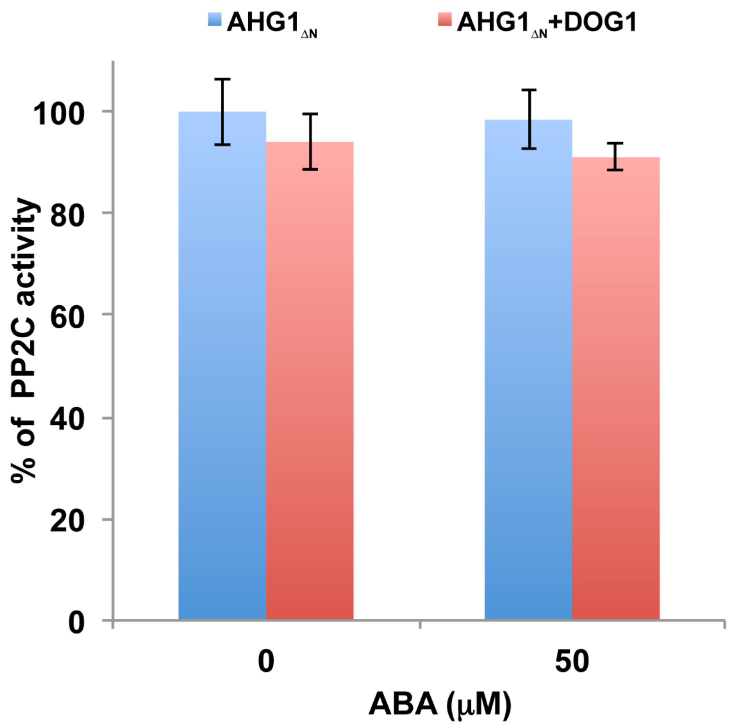

Supplementary Figure 10. Protein phosphatase activity of recombinant truncated AHG1 using an artificial substrate of RRA(pT)VA. The PP2C activities of truncated AHG1$_{\Delta N}$ were measured with or without DOG1 or ABA using a phosphopeptide, RRA(pT)VA (Promega), as a substrate. Error bars show s.d. of three independent experiments.

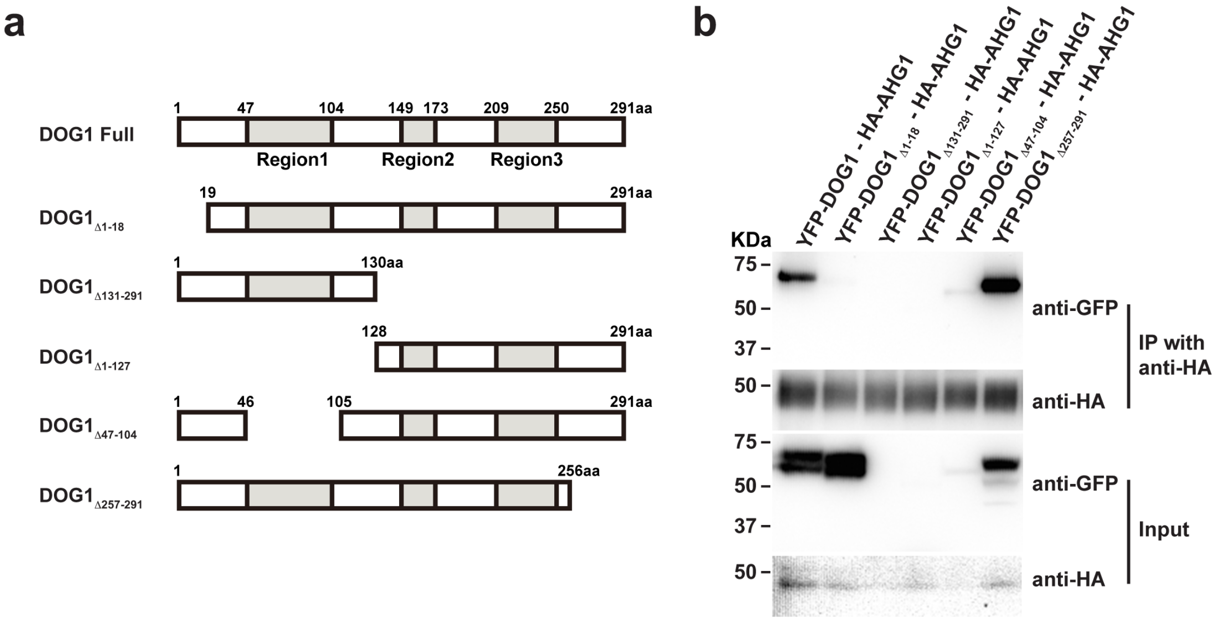

Supplementary Figure 11. Physical interaction of deleted forms of DOG1 with AHG1. (**a**) Delineation of DOG1 and deleted forms of DOG1. The three conserved regions among DOG1/DOGLs are shown gray. (**b**) Interaction of deleted forms of DOG1 with AHG1. HA-AHG1 co-immunoprecipitates with deleted forms of YFP-DOG1. Total protein extracts from transformed *N. benthamiana* leaves were harvested 5 days after inoculation. After co-immunoprecipitation using anti-HA matrix, input and immunoprecipitated samples were detected with anti-GFP and anti-HA antibodies.

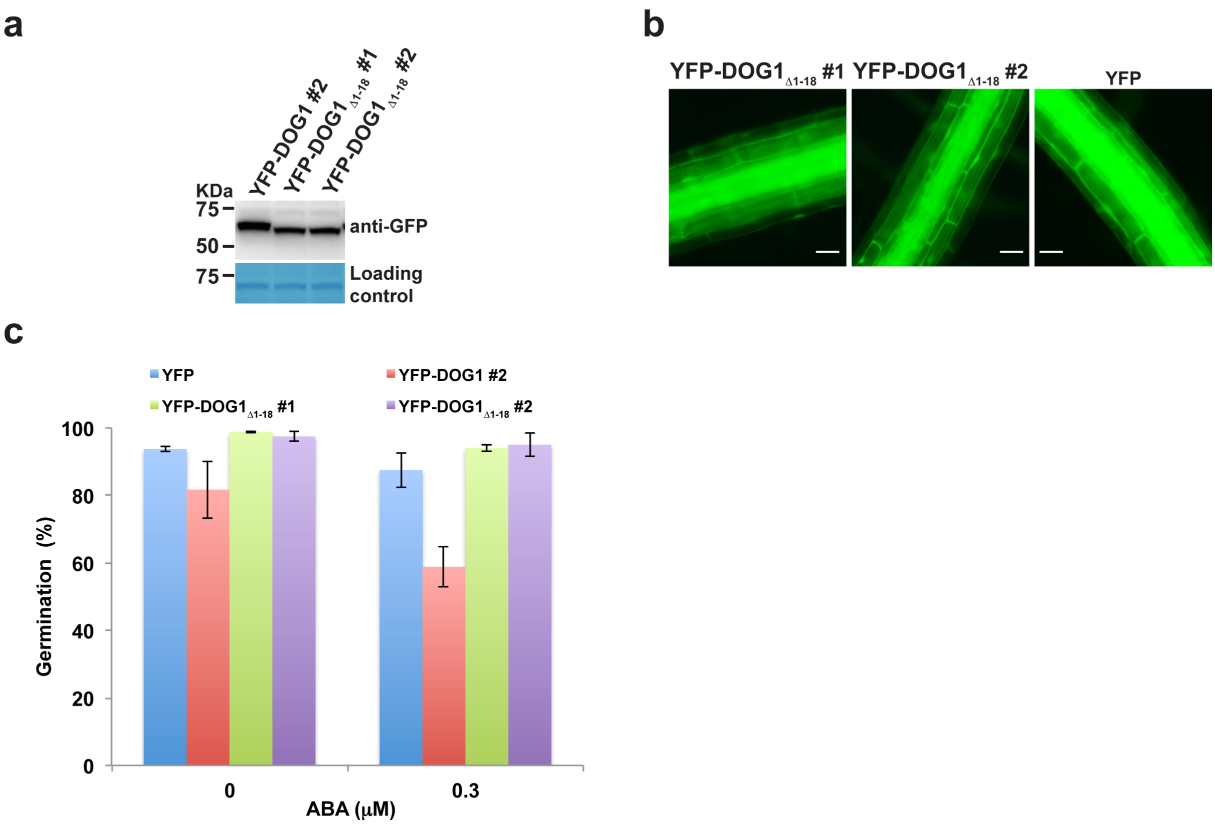

Supplementary Figure 12. YFP-DOG1$_{\Delta 1\text{-}18}$ox lines show the similar germination efficiency to the YFP control line. (**a**) Western blot analysis of YFP-DOG1 and YFP-DOG1$_{\Delta 1\text{-}18}$ protein levels in *Arabidopsis* plants overexpressing YFP-DOG1 and YFP-DOG1$_{\Delta 1\text{-}18}$. Total protein was isolated from seedlings grown on MS plate for 7 days. A nonspecific band stained at approx. 70 kDa is used as a loading control. (**b**) The subcellular localization of *Arabidopsis* plants overexpressing YFP- YFP-DOG1$_{\Delta 1\text{-}18}$ and YFP. Scale bars, 20μm. (**c**) Germination efficiencies of overexpressing YFP-DOG1$_{\Delta 1\text{-}18}$, YFP-DOG1 and control YFP lines were treated with or without 0.3 μM ABA at 3 days after stratification. Error bars show s.d. of three independent experiments using the same seed batch.

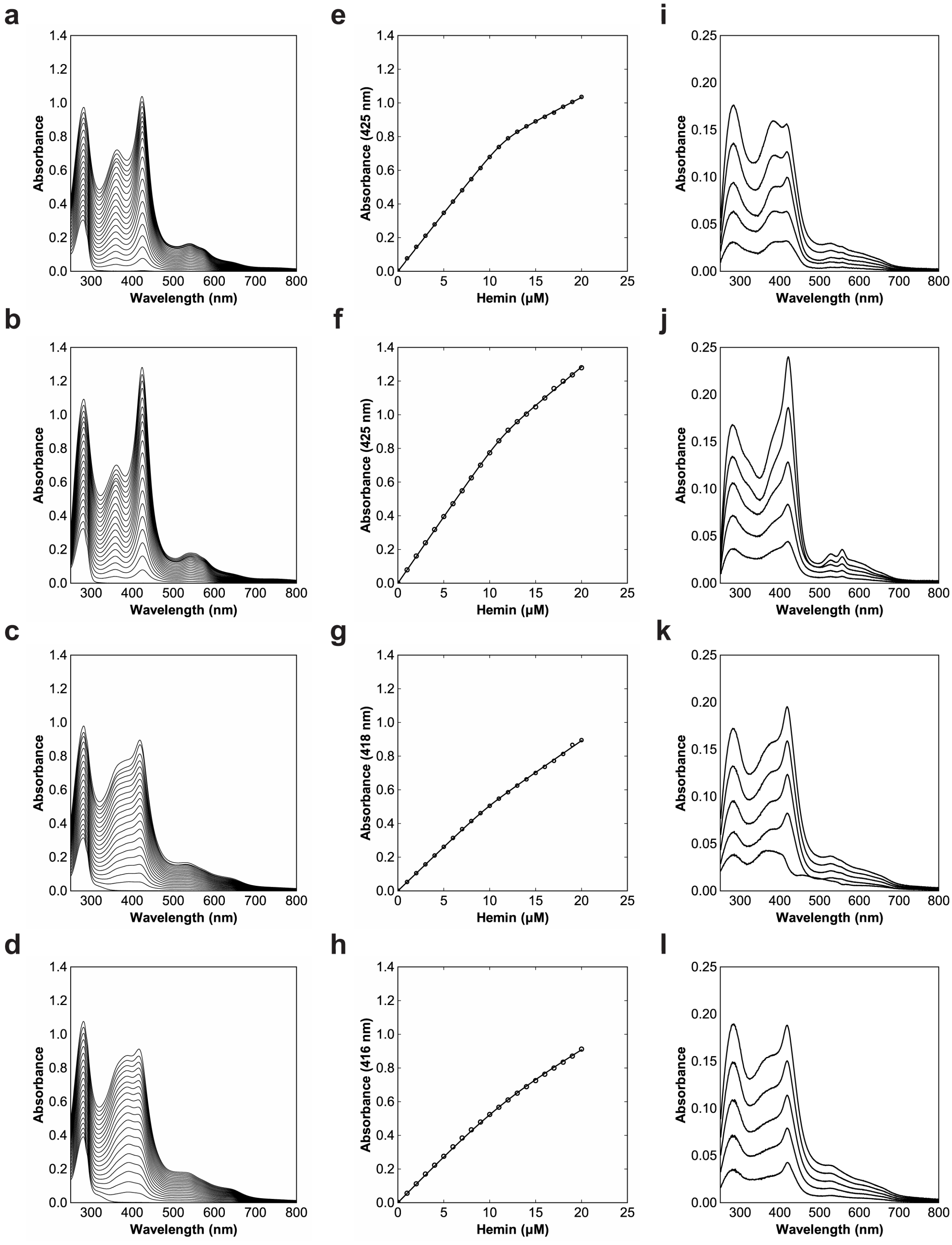

Supplementary Figure 13. Heme binding properties of DOG1 and its mutants. (**a-d**) Electronic absorption spectra, (**e-h**) the absorbance at the selected wavelength plotted as a function of hemin concentration, and (**i-l**) Difference spectra generated by subtracting absorption spectra of hemin concentrations of 15, 16, 17, 18, 19 μM from that of 20 μM for (a,e,i) untagged DOG1 (9.4 μM), (b,f,j) N-terminally His_6-tagged DOG1 (10 μM), (c,g,k) N-terminally His_6-tagged $DOG1^{H245A}$ (9.7 μM), (d,h,l) N-terminally His_6-tagged

DOG1^{H245AH249A} (12 μM). Nonlinear curve fittings of experimental data shown in (e-h) with 1:1 stoichiometry as described in the Methods provided dissociation constants, K_d, of 59 nM for untagged DOG1, 84 nM for His_6-tagged DOG1, 129 nM for His_6-tagged DOG1^{H245A}, and 918 nM for His_6-tagged DOG1^{H245AH249A}.

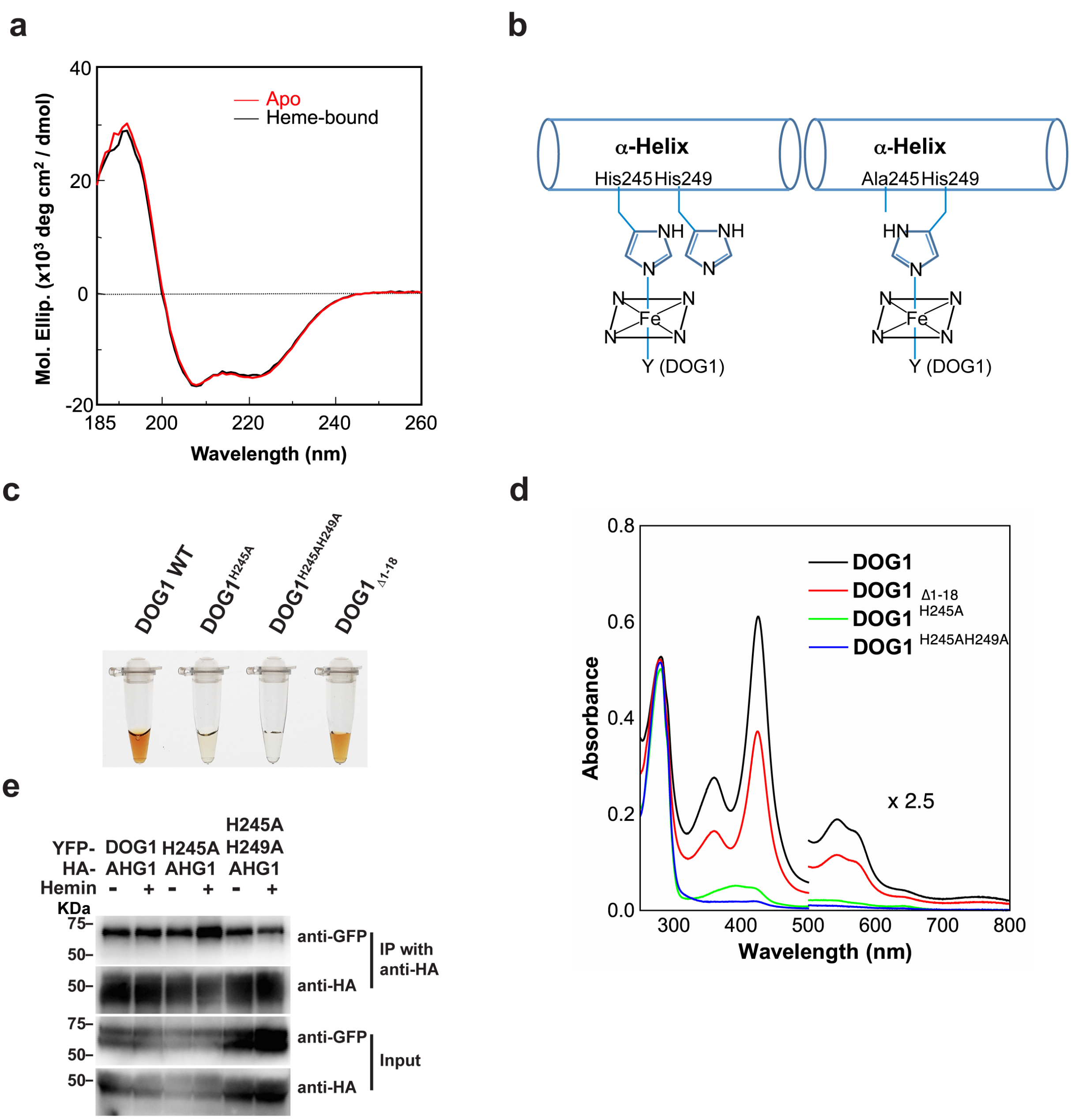

Supplementary Figure 14. Secondary structure of DOG1. (**a**) CD spectra of the N-terminally His$_6$-tagged DOG1 in apo (red) and heme-bound (black) forms in 10 mM sodium phosphate buffer pH 7.5, with 5 mM DTT at protein concentration of 20 µM. (**b**) Schematic representations of hexacoodinate low spin heme in the wild-type DOG1 (left) and the H245A single mutant DOG1 (right). Y represents an undefined axial ligand from DOG1. (**c**) *E. coli* expressed N-terminally His$_6$-tagged DOG1 (29 µM), DOG1^{H245A} (40 µM), DOG1^{H245AH249A} (40 µM) and DOG1$_{\Delta 1\text{-}18}$ (40 µM) for 50-60 h in TB medium (condition II). (**d**) Electronic absorption spectra of the DOG1 (11 µM), DOG1^{H245A} (15 µM), DOG1^{H245AH249A} (15 µM) and DOG1$_{\Delta 1\text{-}18}$ (15 µM) shown in (c). (**e**) Interaction of AHG1 with DOG1, DOG1^{H245A} and DOG1^{H245AH249A}. HA-AHG1 co-immunoprecipitates with YFP-DOG1, YFP- DOG1^{H245A} and YFP- DOG1^{H245AH249A} in a heme-independent manner. Total protein extracts from transformed *N. benthamiana* leaves were harvested 4 days after inoculation and were treated with or without 50 µM hemin for 24 h before harvesting. After

co-immunoprecipitation using anti-HA matrix, input and immunoprecipitated samples were detected with anti-GFP and anti-HA antibodies.

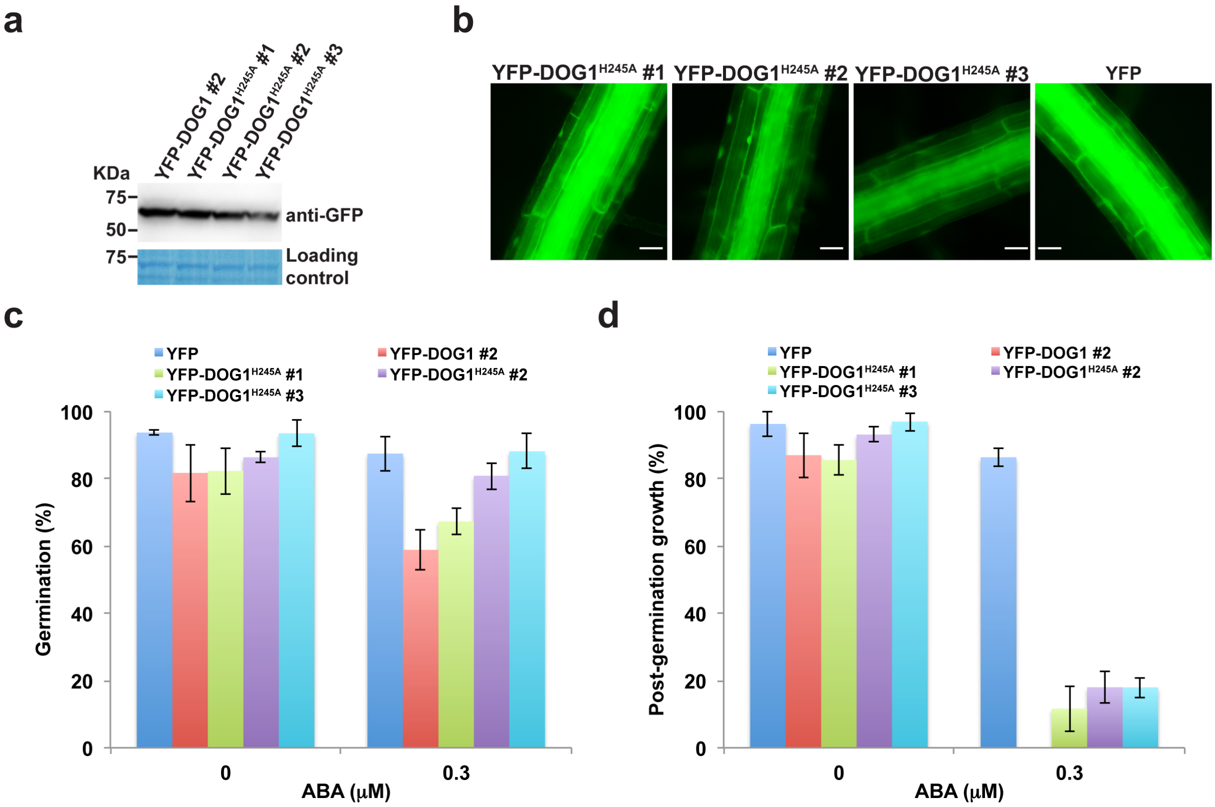

Supplementary Figure 15. YFP-DOG1^{H245A}ox lines show lower germination and post-germination growth efficiencies to the YFP control line. (**a**) Western blot analysis of YFP-DOG1 and YFP-DOG1^{H245A} protein levels in *Arabidopsis* plants overexpressing YFP-DOG1 and YFP-DOG1^{H245A}. Total protein was isolated from seedlings grown on MS plate for 7 days. A nonspecific band stained at approx. 70 kDa is used as a loading control. (**b**) The subcellular localization of *Arabidopsis* plants overexpressing YFP-DOG1^{H245A} and YFP. Scale bars, 20µm. (**c,d**) Germination efficiencies (c) and post-germination growth efficiencies (d) of overexpressing YFP-DOG1^{H245A}, YFP-DOG1 and control YFP lines were treated with or without 0.3 µM ABA at 3 days (c) and 7 days (d) after stratification. Error bars show s.d. of three independent experiments using the same seed batch (c,d).

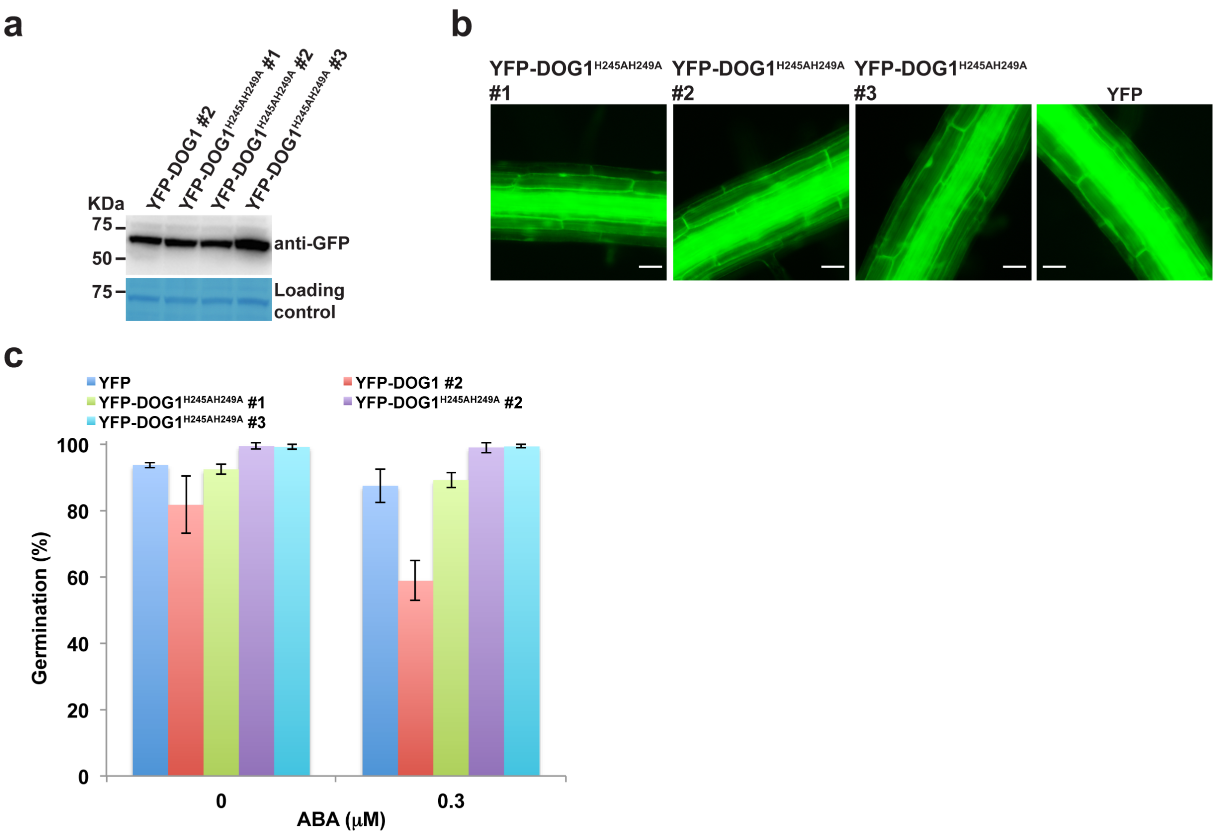

Supplementary Figure 16. YFP-DOG1^{H245AH249A}ox lines show the similar germination efficiency to the YFP control line. (**a**) Western blot analysis of YFP-DOG1 and YFP-DOG1^{H245AH249A} protein levels in *Arabidopsis* plants overexpressing YFP-DOG1 and YFP-DOG1^{H245AH249A}. Total protein was isolated from seedlings grown on MS plate for 7 days. A nonspecific band stained at approx. 70 kDa is used as a loading control. (**b**) The subcellular localization of *Arabidopsis* plants overexpressing YFP-DOG1^{H245AH249A} and YFP. Scale bars, 20μm. (**c**) Germination efficiencies of overexpressing YFP-DOG1^{H245AH249A}, YFP-DOG1 and control YFP lines were treated with or without 0.3 μM ABA at 3 days after stratification. Error bars show s.d. of three independent experiments using the same seed batch.

Fig. 1b

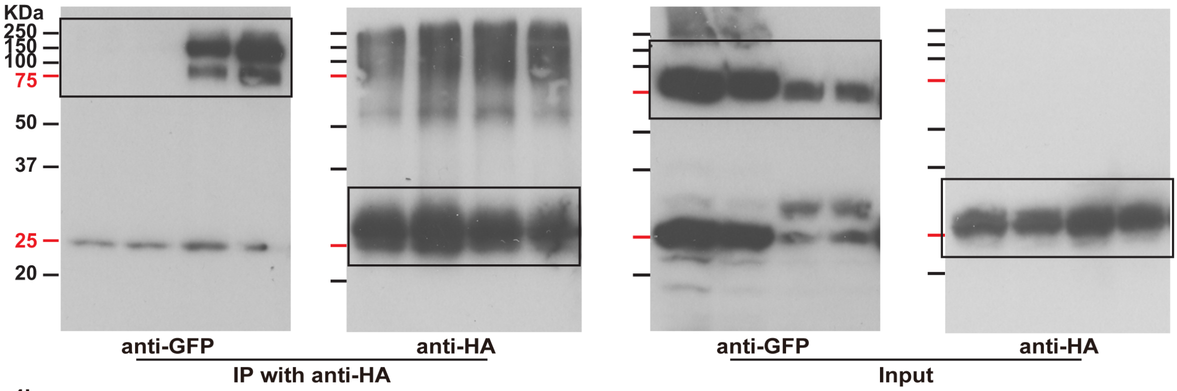

Fig. 4b

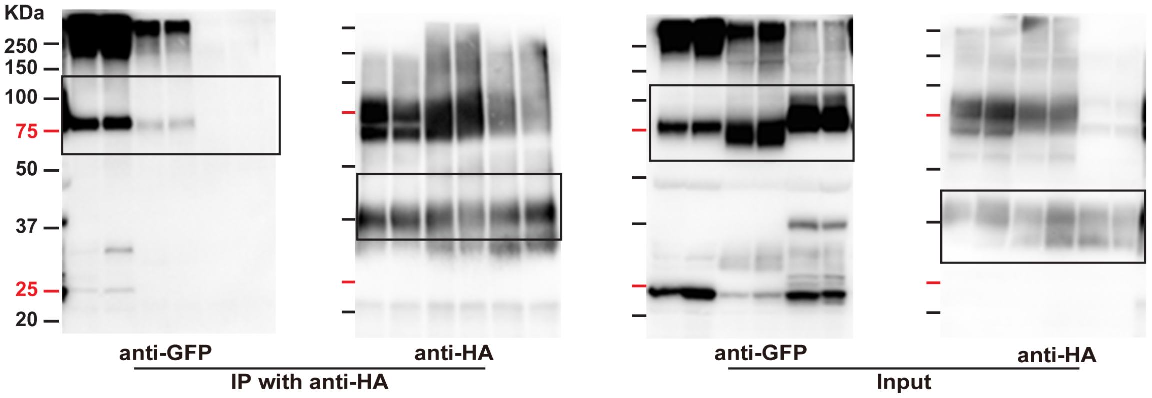

Fig. 6b

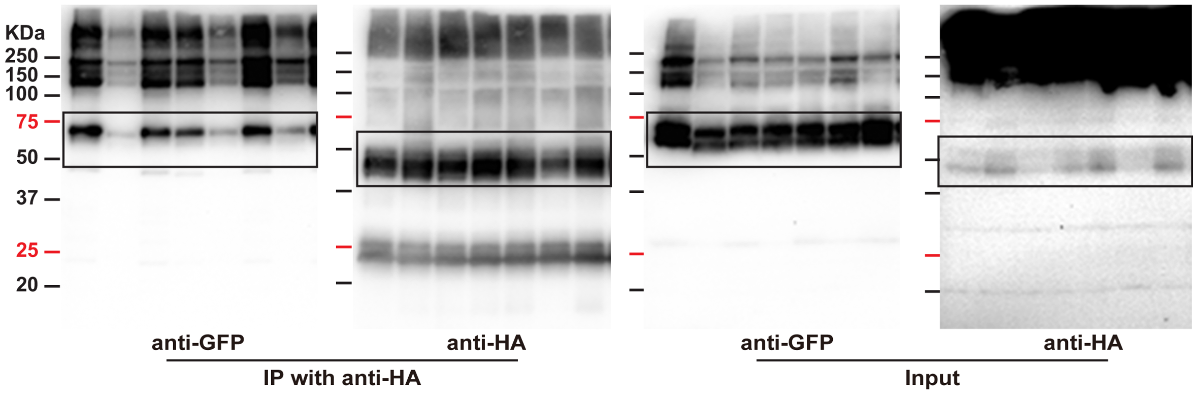

Supplementary Fig. 4a

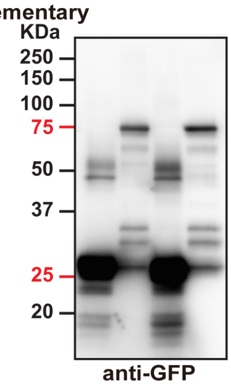

Supplementary Fig. 4b

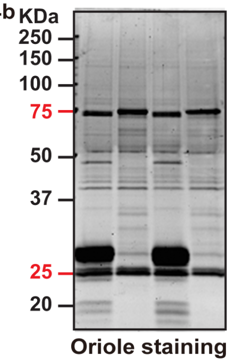

Supplementary Fig. 5a

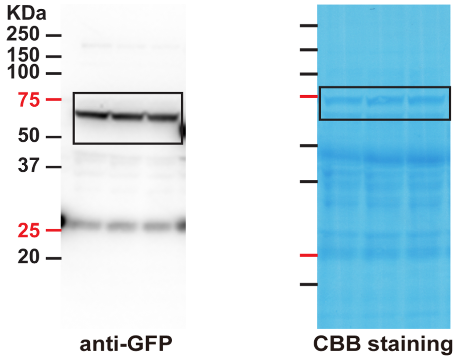

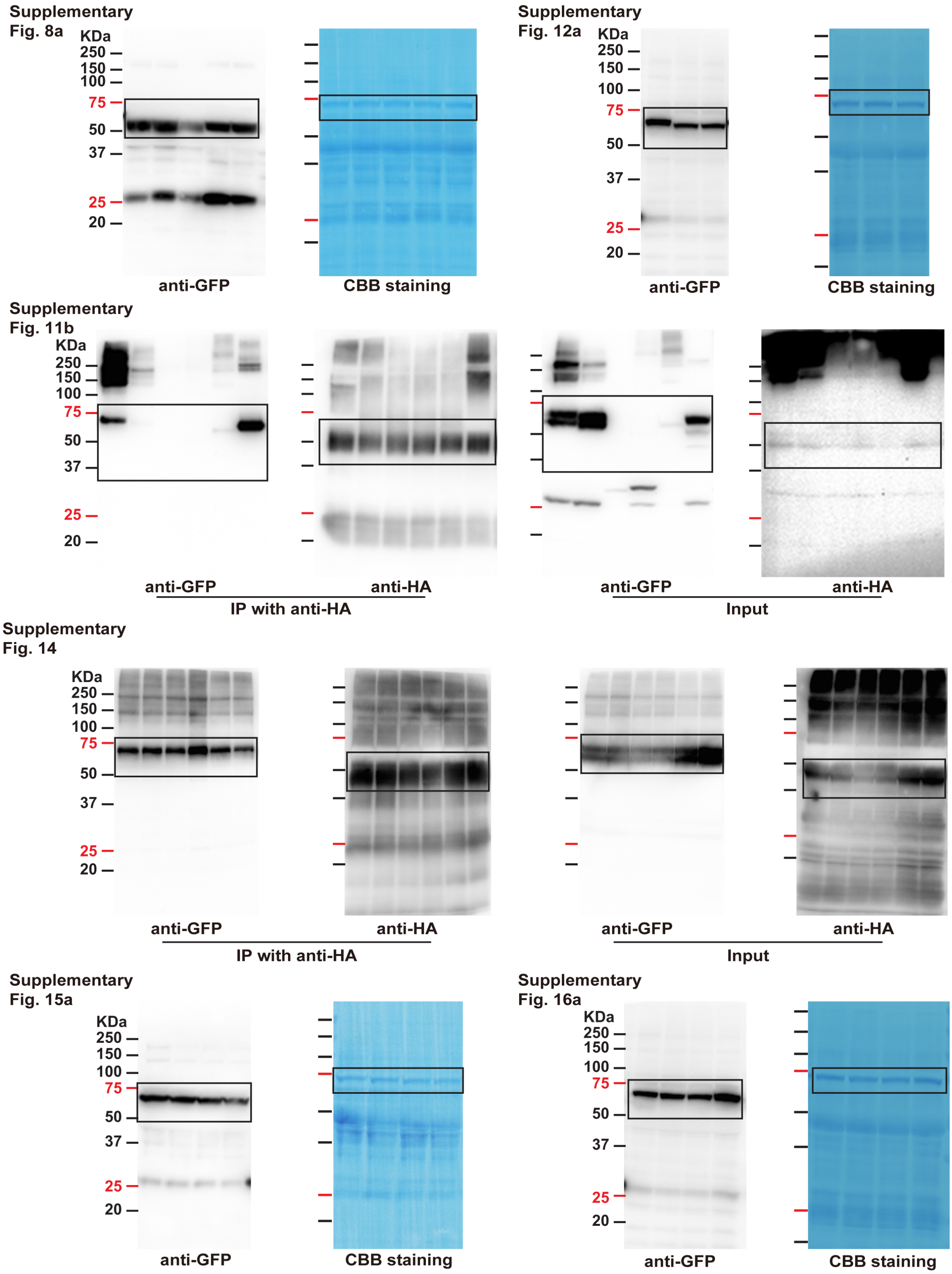

Supplementary Figure 17. Full scan data of immunoblots and gel.

Supplementary Fig. 4d

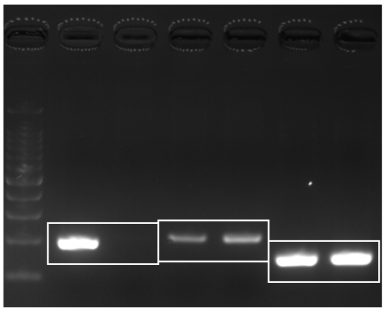

Supplementary Fig. 9a

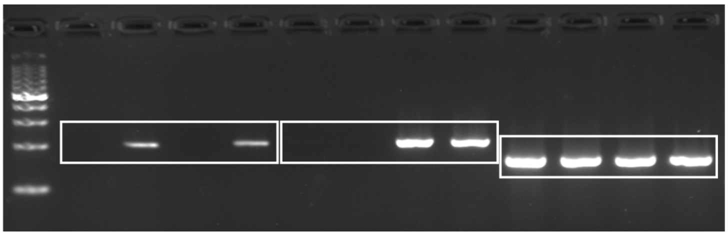

Supplementary Figure 18. Full scan data of gel.

Supplementary Table 1. The sequence coverage of AHG1-interacting proteins validated by yeast-two-hybrid assay.

AGI	Symbol	Sequence coverage (%)						
		Ex1	Ex2	Ex3	Ex4	Ex5	Ex6	Average sequence coverage
AT5G51760	AHG1	74.5	90.4	78.4	75.0	61.5	83.9	77.3 ± 9.8
AT1G13740	AFP2	-	-	6.6	13.2	10.9	-	10.2 ± 3.4
AT1G15750	TPL	-	2.0	4.7	3.5	-	3.4	4.1 ± 1.1
AT2G19520	FVE	-	9.7	-	-	7.7	17.6	7.7± 5.2
AT5G45830	DOG1	-	17.2	30.2	21.6	-	-	25.9 ± 6.6

YFP-AHG1ox plants were treated with (Ex4 to Ex6) or without (Ex1 to Ex3) ABA.

Supplementary Table 2. The numbers of unique and total peptides and spectrum count of AHG1-interacting proteins validated by yeast-two-hybrid assay.

		Unique peptides / Total peptides (Spectrum count)						
AGI	Symbol	Ex1	Ex2	Ex3	Ex4	Ex5	Ex6	Average spectrum count
AT5G51760	AHG1	71/71 (187)	173/173 (387)	107/107 (302)	123/123 (335)	54/54 (151)	124/124 (325)	281.2 ± 91.9
AT1G13740	AFP2	0	0	2/2 (2)	3/3 (3)	2/2 (2)	0	2.3 ± 0.6
AT1G15750	TPL	0	0/2 (2)	0/3 (3)	2/2 (2)	0	2/2 (2)	2.3 ± 0.5
AT2G19520	FVE	0	2/3 (3)	0	0	3/3 (3)	6/6 (6)	4.0 ± 1.7
AT5G45830	DOG1	0	2/2 (2)	3/3 (3)	4/4 (4)	0	0	3.0 ± 1.0

YFP-AHG1ox plants were treated with (Ex4 to Ex6) or without (Ex1 to Ex3) ABA.

Supplementary Table 3. Parameters derived from fitting[a] the electronic absorption data of hemin titration experiments.

Protein	K_d (nM)	εDH[b] ($mM^{-1}cm^{-1}$)	εH[c] ($mM^{-1}cm^{-1}$)	x[d]
Untagged DOG1	58.8	79.4	27.4	0.81
His_6-DOG1	84.4	84.1	44.7	0.88
His_6-DOG1^{H245A}	129	52.4	36.9	1.0
His_6-DOG1^{H245AH249A}	918	57.1	37.4	1.0

[a] Experimental data shown in Supplementary Fig. 13e-h were fit to a 1:1 stoichiometric heme binding as described in the Methods.
[b] Extinction coefficient of the protein-bound heme at 425 nm for untagged and His_6-tagged DOG1, at 418 nm for His_6-tagged DOG1^{H245A}, and 416 nm for DOG1^{H245AH249A}.
[c] Extinction coefficient of the free hemin at 425 nm for untagged and His_6-tagged DOG1, at 418 nm for His_6-tagged DOG1^{H245A}, and 416 nm for DOG1^{H245AH249A}.
[d] Fraction of the active hemin which can be incorporated into protein.

Supplementary Table 4. List of oligonucleotides used in this study.

Name	Sequence	Purpose	Information	Ref
ahg1-1 F	CGGTATACGATGGCCACGGCGGAGCT	mutation detection	dCAPS *Sac*I	Ref. 4
ahg1-1 R	CTCCTCTTCGAGGTTTTGCTCTA	mutation detection		
ahg3-1 F	TGGGATGGAGCTAGGGTTCATG	mutation detection	dCAPS *Bsp*HI	Ref. 5
ahg3-1 R	ATCACTCGCCAAGATCAAACAC	mutation detection		
hai1-2 F	TTGAAACAACGTCCCAAAGAC	mutation detection	SALK_1082282	Ref. 6
hai1-2 R	TTTCACTATTGCGGCGTTTAC	mutation detection		
aip1-1 F	CAGATTTATCCTCCTCCTCCG	mutation detection	SALK_090738	Ref. 6
aip1-1 R	TCTTTGCAAATGTTTTAAACACG	mutation detection		
hai3-1 F	TCACGCATAGCACAAAACAAG	mutation detection	SALK_033011	Ref. 6
hai3-1 R	TTCTCCCAATCTTCATGCATC	mutation detection		
dog1-2 F	TCTTTAGGCTCGTTTATGCTTTGTGGAGCT	mutation detection	dCAPS *Sac*I	Ref. 7
dog1-2 R	ACCGTACTGACTACCGAACC	mutation detection		
dog1-3 R	ATTTCCTCGGAAAGCACGTA	mutation detection	SALK_000867	
dog1-3 R	GGAGAAACTGAGTCACACGGATCTC	mutation detection		
AHG1 F1	AAACCGAATTTGTGCAAACC	RT-PCR		
AHG1 R1	CCATTTCCTCTCCACGACAT	RT-PCR		
DOG1-overall-F	GAGCTGATCTTGCTCACCGATGTAG	RT-PCR		Ref. 7
DOG1-overall-R	CCGCCACCACCTGAAGATTCGTAG	RT-PCR		
18S F	AAACGGCTACCACATCCAAG	RT-PCR		
18S R	CCTCCAATGGATCCTCGTTA	RT-PCR		

Supplementary References

1. Larkin, M.A. *et al.* Clustal W and Clustal X version 2.0. *Bioinformatics* **23**, 2947-2948 (2007).
2. Drozdetskiy, A., Cole, C., Procter, J. & Barton, G.J. JPred4: a protein secondary structure prediction server. *Nucleic Acids Res.* **43**, W389-W394 (2015).
3. Kelley, L.A., Mezulis, S., Yates, C.M., Wass, M.N. & Sternberg, M.J. The Phyre2 web portal for protein modeling, prediction and analysis. *Nat. Protoc.* **10**, 845-858 (2015).
4. Nishimura, N. *et al. ABA-Hypersensitive Germination1* encodes a protein phosphatase 2C, an essential component of abscisic acid signaling in Arabidopsis seed. *Plant J.* **50**, 935-949 (2007).
5. Yoshida, T. *et al. ABA-hypersensitive germination3* encodes a protein phosphatase 2C (AtPP2CA) that strongly regulates abscisic acid signaling during germination among Arabidopsis protein phosphatase 2Cs. *Plant Physiol.* **140**, 115-126 (2006).
6. Bhaskara, G.B., Nguyen, T.T. & Verslues, P.E. Unique drought resistance functions of the *Highly ABA-Induced* clade A protein phosphatase 2Cs. *Plant Physiol.* **160**, 379-395 (2012).
7. Nakabayashi, K. *et al.* The time required for dormancy release in *Arabidopsis* is determined by DELAY OF GERMINATION1 protein levels in freshly harvested seeds. *Plant Cell* **24**, 2826-2838 (2012).
