# Supplementary material for: Control of seed dormancy and germination by DOG1-AHG1 PP2C phosphatase complex via binding to heme
Source: Nat Commun. 2018 Jun 6;9:2132. doi: 10.1038/s41467-018-04437-9 (PMC5989226; doi:10.1038/s41467-018-04437-9)
Supplement: Supplementary file 3 — Description of Additional Supplementary Files [file 41467_2018_4437_MOESM3_ESM.pdf]

Description of Additional Supplementary Files

Supplementary Data 1. List of AHG1-interacting protein candidates in the YFP-AHG1ox plants derived from LC-MS/MS data.

Supplementary Data 2. List of YFP-interacting protein candidates in the YFPox plants derived from LC-MS/MS data.
